# Analytical approaches for investigation of partially identified leachables in ENDS

**DOI:** 10.3389/fchem.2026.1888746

**Published:** 2026-08-05

**Authors:** E. Diane Wallace, Cameron Smith, Aliya Al-Habsha, I. Gene Gillman

**Affiliations:** Juul Labs, Washington, DC, United States

**Keywords:** e-cigarette, electronic cigarette, ENDS, extractables, leachables, nicotine

## Abstract

Electronic nicotine delivery systems (ENDS), or e-cigarettes, are battery-powered devices that produce an aerosol for inhalation by heating e-liquid. E-liquid is typically composed of propylene glycol (PG), vegetable glycerin (VG), nicotine salt, and is contained within a container closure system (CCS), such as a prefilled pod or disposable device. These container closure systems are commonly manufactured with materials such as plastics, elastomers, glass, metals, etc., which have the potential to leach unintended chemical compounds into the e-liquid. Given that these leachable compounds may transfer to the aerosol during consumer use, the United States Food and Drug Administration (U.S. FDA) requires ENDS manufacturers to conduct extractable and leachable (E&L) studies to assess potential safety risks. One significant challenge in leachable studies for ENDS products is that e-liquid naturally changes over time, forming trace degradants and reaction products despite the materials used in the container closure system. Because leachable studies often utilize non-targeted, highly sensitive, analytical methods to investigate changes over time, the origin of a compound observed during the aging process can be difficult to ascertain when compounds are not observed in extractable studies. In this work, a case study was presented in which five partially identified, data-deficient, leachable compounds were reported following 12 months of room temperature storage of unflavored e-liquid in a JUUL2 pod container closure system that were not observed in extractable studies or in initial unaged samples. An experiment was conducted where unflavored e-liquid was artificially aged outside the JUUL2 pod, and all five of the data-deficient compounds were observed. Additional experiments using isotopically labelled primary ingredients confirmed that four of the five studied organic compounds were derived from nicotine, and the fifth was derived from benzoic acid. The analytical approaches outlined in this work provided a technique to determine whether select organic compounds observed in long term leachable studies were derived from the container closure system.

## 1 Introduction

Extractable and leachable (E&L) studies are a critical component of product safety assessment, designed to identify chemical compounds that can migrate from a product’s packaging or pod/device components—collectively known as the container closure system (CCS)—into the final product. Extractables are compounds drawn out under aggressive, worst-case laboratory conditions (e.g., harsh solvents, elevated temperatures). Leachables are compounds that migrate into the product under normal conditions of storage and use. Electronic Nicotine Delivery Systems (ENDS), or e-cigarettes, manufacturers are required to conduct E&L studies as part of their premarket tobacco product application (PMTA) to ensure extractables and leachables are screened for potential hazards and appropriately risk assessed prior to consumer use (USFDA, 2023). While the FDA has not issued formal E&L guidance specifically for ENDS, FDA’s 2020 memorandum (USFDA, 2020) recommends that manufacturers leverage published literature (Ball et al., 2007; Norwood et al., 2008) and established standards from the pharmaceutical industry to design and conduct E&L studies. This includes best practices from the International Council for Harmonisation of Technical Requirements for Pharmaceuticals for Human Use (ICH) for Orally Inhaled and Nasal Drug Products (OINDPs) (International Council for Harmonisation of Technical Requirements for Pharmaceuticals for Human Use, 2019), guidance from the Center for Drug Evaluation and Research (CDER) (USFDA, 2002), and relevant chapters from the United States Pharmacopeia (USP) (USP, 2024a; USP, 2024b). The recommendation to consult OINDP guidance is predicated on material similarities (e.g., plastics, elastomers, metals) between ENDS and OINDPs, which often yield comparable extractable profiles, including additives, pigments, plasticizers, and curing agents (Jenke, 2017; Jenke et al., 2013). However, ENDS utilize primary e-liquid ingredients propylene glycol [PG] and vegetable glycerin [VG] as the carrier housed in the CCS in which worst case, i.e., harsh organic solvent extractions at elevated temperatures, extractable results often provide minimal overlap with results obtained in long term leachable studies conducted at ambient storage conditions. This makes sense because PG and VG are relatively mild solvents housed in relatively inert materials and commercial packaging practices are efficient at limiting environmental conditions, e.g., oxygen, light, moisture, both of which influence leaching. Inorganic leachable compounds have been studied heavily in literature, as most modern ENDS products utilize nicotine salts. These salts create an acidic environment conducive to the leaching of metals from the heating element, or coil, into the e-liquid (Alcantara et al., 2023). It is important to note, however, that the FDA currently classifies the wick and coil as device components rather than part of the primary CCS. Current recommendations for leachable studies (USFDA, 2023) include both targeted analysis for compounds identified during extractable screening and non-targeted analysis to detect potential unidentified migrants. Non-targeted approaches can present analytical challenges for aged ENDS products, because the primary e-liquid constituents are known to form degradants, unrelated to the CCS, in e-liquid over time (Erythropel et al., 2019; Flora et al., 2016; Jaegers et al., 2021). These degradants can be difficult to distinguish from true leachables derived from the CCS when not observed in extractable studies nor initial unaged samples. To address this challenge, this case study investigates the source of five data-deficient organic compounds reported from a long-term simulated leachable study from a closed-pod based ENDS product, JUUL2 pods filled with unflavored e-liquid containing primary ingredients [PG, VG, nicotine, and benzoic acid] that were not observed in either extractable studies nor initial unaged samples. The primary goal of the study was to determine if the observed organic compounds were true leachables from the CCS, or simply reaction products of primary e-liquid ingredients. To investigate this, experiments were performed by artificially aging (i.e., heating) unflavored e-liquid to determine whether compounds could form outside of the JUUL2 pod. If compounds formed outside the JUUL2 pod, additional experiments were performed using isotopically labelled primary ingredients (PG, VG, benzoic acid and nicotine) to determine the source of the observed compounds. The approach utilized herein provides a strategy to determine whether compounds are potential leachables that arise from the CCS or derived exclusively from primary ingredients in e-liquid unrelated to the CCS.

## 2 Methods

### 2.1 Case study background

A simulated leachable study was conducted at WuXi AppTec., Inc (St. Paul, Mn) on JUUL2 pods filled with unflavored e-liquid (PG, VG, nicotine, and benzoic acid) containing 5.0% (w/w) nicotine stored at room temperature storage conditions (22 °C and 60% RH) for approximately 1 year. The unflavored e-liquid utilized the same base ingredients as commercial JUUL2 pod formulations, however, unflavored JUUL2 pods are not commercially available. The samples were analyzed by non-targeted gas chromatography-mass spectrometry (GC-MS) and liquid chromatography-mass spectrometry (LC-MS) in both positive and negative electrospray ionization (ESI) mode to cover a wide range of volatile, semi-volatile and non-volatile organic compounds. An analytical evaluation threshold (AET) of 0.25 µg/pod with an uncertainty factor of 3 for GC-MS, and 0.15 µg/pod with an uncertainty factor of 5 for LC-MS, was derived from the threshold of toxicological concern (TTC) value of 1.5 µg/day, assuming an exposure of two JUUL2 pods/day.

In addition to e-liquid analysis, aerosol was generated using a JUUL2 device with JUUL2 pods filled with unflavored e-liquid aged for approximately 1 year, using a previously published aerosol collection method described in Smith et al. (2023). An additional in-house intense puffing regime (intense puffing) was also performed using a 110 mL puff volume, 6 s puff duration, and 30 s puff intervals. JUUL2 devices were fully charged prior to aerosol generation and collection, in which 105 puffs were collected using the ISO 20768 standard puffing regime (ISO puffing) and 81 puffs were collected using the in-house intense puffing regime. For each electrostatic precipitation aerosol collection port, four JUUL2 pods were collected, equating to approximately 4 g of collected aerosol. A total of 16 replicates were performed, and all replicates were pooled together for a final sample volume of 40 mL. The final pooled aerosol samples (one ISO puffing pooled sample and one intense puffing pooled sample) were stored at freezer conditions and shipped to WuXi AppTec., Inc. to be analyzed in triplicate alongside the e-liquid samples.

All studies, except for the simulated leachable study discussed above, were performed at the Juul Labs Regulatory Chemistry Laboratory (JLRCL, Durham, NC). Specific analytical method details regarding analyses performed by WuXi AppTec., Inc. during long-term leachable studies are proprietary, however, all follow-up analyses performed by JLRCL are described in Sections 2.2 and 2.3.

### 2.2 Sample preparation

Approximately 1 mL of unflavored e-liquid (50:50 PG:VG, 5% nicotine, 3.8% benzoic acid) was heated at 50 °C for 2 days in a small 12 mL amber glass vial, then extracted using 3 mL of dichloromethane (DCM). The organic layer was transferred to a separate vial. The sample layer was extracted an additional two times with 3 mL of DCM, combining the pooled organic layer to provide a final extraction volume of 9 mL. An aliquot of extract was taken for analysis. Internal standards [butylated hydroxytoluene (MP Biomedicals Santa Ana, CA), diphenylamine (Fisher Scientific Waltham, MA), dodecalactam (Sigma-Aldrich St. Louis, MO), and tributylacetyl citrate (MilliporeSigma Burlington, MA)] were spiked into the final extracts at 2 μg/mL to assess retention times for method comparison between laboratories.

For isotopic labelling experiments, deuterated analytical standards, benzoic-D5 acid [Product No. D-0168], propylene glycol-D6 [Product No. D-1507], glycerin-D5 [Product No. D-1387] and nicotine-D4 [Product No. D-5098] (CDN Isotopes, Quebec, Canada), were exchanged, one at a time, for each primary ingredient in the unflavored e-liquid. Each e-liquid was formulated by weighing approximately 0.077 g of benzoic acid into a 4 mL vial then adding approximately 0.911 g of propylene glycol. All vials were sonicated until benzoic acid was dissolved. Approximately 0.911 g of glycerol and approximately 0.100 g of neat nicotine were added and mixed well. A labelled e-liquid was formulated for each primary ingredient (PG, VG, benzoic acid, and nicotine) as well as an unlabeled control e-liquid for a total of five different formulations. A total of approximately 1.9 g of e-liquid was created for each sample.

All isotopically labelled unflavored e-liquid samples, and an unlabeled unflavored e-liquid control, were heated at 50 °C for 7 days and extracted in the same manner discussed above. Heating time was increased from 2 to 7 days to give additional time for reactions to form and increase concentration, as the compounds were in low abundance in the e-liquid heated at 50 °C for 2 days. In accordance with ASTM F1980-21 (2021), accelerated aging for 2 and 7 days at 50 °C is equivalent to approximately 11 and 40 days at 25 °C when a default Q_10_ value of 2.0 is used for simulation.

### 2.3 GC-HRMS analysis

Analyses of each sample, one independent replicate for each experiment discussed in Section 2.2, were conducted on a Thermo TRACE 1610 gas chromatograph coupled to a Thermo Exploris 240 mass spectrometer. Chromatographic separation was achieved using an Agilent DB-1MS UI capillary column (30 m × 0.25 mm, 0.25 µm film thickness). The temperature of the oven was programmed from 40 °C to 300 °C at a rate of 10 °C/min, with initial and final hold times of one and 10 minutes, respectively. The inlet and interface temperatures were 210 °C and 280 °C, respectively. Helium, at a flow rate of 1.0 mL/min (constant flow), was used as the carrier gas. Samples (1.0 µL) were injected using pulsed splitless mode at 20 psi, with a purge flow of 30 mL/min at 0.7 min. Electron impact (EI) mass spectrometry was performed at 70 eV, and ion source temperature was maintained at 230 °C. Data acquisition was performed after a 4.5-min solvent delay, with full scan (35–650 m/z), 15,000 resolving power, AGC target standard, and a maximum inject time of 100 ms. Data analysis and visualization was conducted using Xcalibur 4.5.

## 3 Results and discussion

Table 1 shows the GC-MS results for five partially identified organic compounds (labelled A through E) reported by WuXi AppTec., Inc., observed in the analysis of e-liquid removed from 12-month ambient aged JUUL2 pods that were not present in extractable studies nor the initial, unaged sample. Reported compounds lacked tentative structures and CAS numbers, and justification for the partial identifications provided from WuXi AppTec., Inc. were based on general fragmentation patterns characteristic of aromatic compounds, benzene (77 and 91 *m/z*) and benzyl amines (106 *m/z*).

**TABLE 1:** Chemical information for reported partially identified leachable compounds by GC-MS in unflavored e-Liquid removed from aged JUUL2 pods.

Compound ID	Reported compound information^1^	ID confidence	Relative retention time (min)^1^ ^,^ ^2^	Estimated concentration in e-Liquid^1^ (mcg/pod)	Estimated concentration in aerosol^1^ (mcg/pod)		Observed m/z values^1^
					Intense puffing	ISO puffing	
Compound A	Alkylbenzene related compound	Partial	0.74	0.57 ± 0.09	1.2 ± 0.2	0.94 ± 0.07	77, 91, 105, 119, 135
Compound B	Alkylbenzene related compound	Partial	1.40	0.57 ± 0.02	0.76 ± 0.03	0.72 ± 0.07	91, 118, 175
Compound C	Alkylbenzene related compound	Partial	1.41	2.0 ± 0.0	2.1 ± 0.1	2.1 ± 0.2	91, 118, 175
Compound D	Alkylbenzeneamine related compound	Partial	1.07	3.5 ± 0.2	2.4 ± 0.3	2.7 ± 0.2	78, 84, 106, 134, 161, 173, 190
Compound E	Alkylbenzeneamine related compound	Partial	1.42	2.1 ± 0.2	Not detected	Not detected	130, 156, 171, 217, 274

1 Information provided by WuXi AppTec., Inc.

2 Calculated based on retention time of BHT.

**FIGURE 1:**
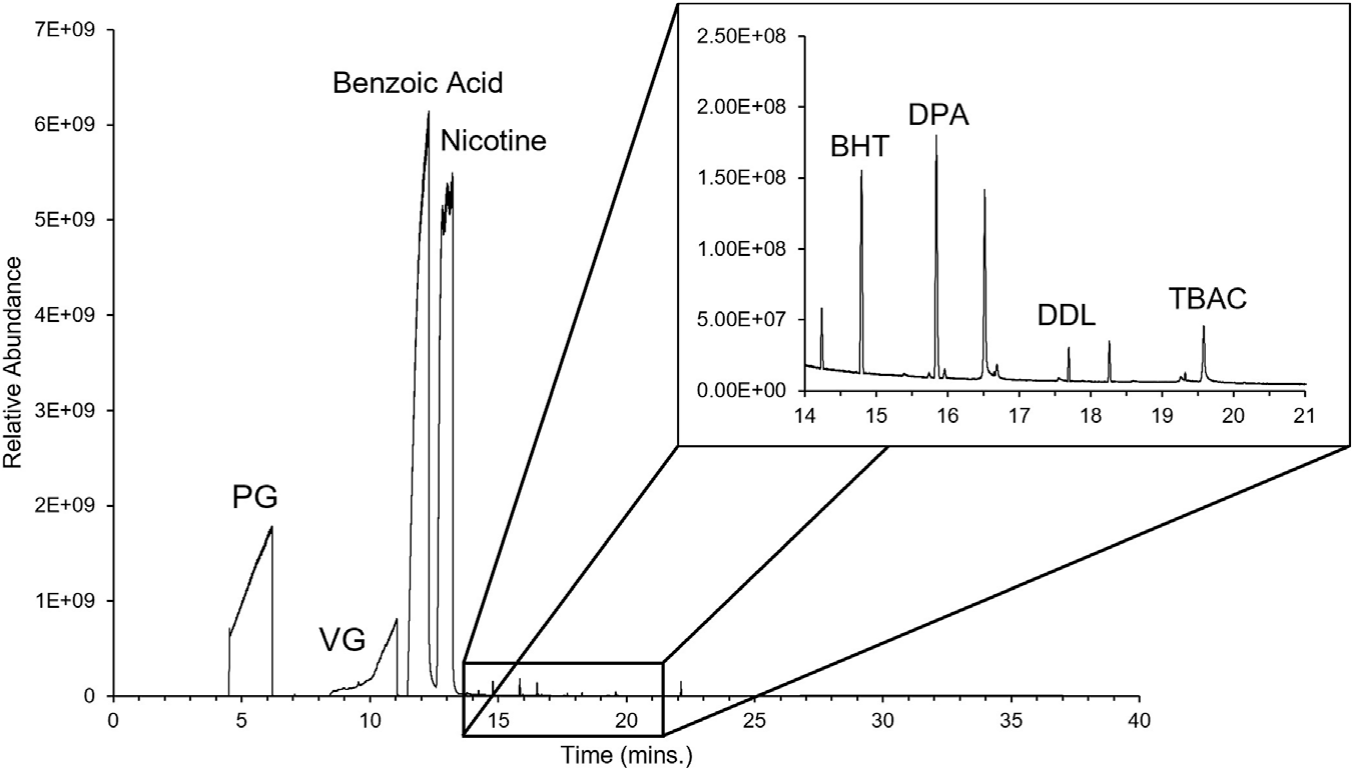
GC-MS chromatogram of extracted e-liquid, heated for 2 days at 50 °C outside the JUUL2 pod prior to extraction, spiked with 2 μg/mL of internal standards butylated hydroxytoluene (BHT), diphenylamine (DPA), dodecalactam (DDL), and tributyl acetylcitrate (TBAC).

Simulated aging by heating the unflavored e-liquid separate from the JUUL2 pod at JLRCL was successful at reproducing all five partially identified compounds from the simulated leachable study conducted at WuXi AppTec., Inc. This result provided evidence their origin was formulation related, not leachable compounds from the JUUL2 pod container closure system. Figure 1 shows the GC-HRMS chromatographic profile of the heated, unflavored e-liquid sample after 2 days at 50 °C. The five compounds, A-E, were confirmed by matching their mass spectra and relative retention times to the WuXi AppTec., Inc. reported GC-MS data. To ensure an accurate comparison between the two analyses, internal standards butylated hydroxytoluene (BHT), diphenylamine (DPA), dodecalactam (DDL), and tributyl acetylcitrate (TBAC) were used to confirm system compatibility; BHT was used to calculate relative retention time (RRT). It’s worth noting that 2 days at 50 °C, simulating 11 days of ambient storage, was chosen to provide a quick assessment as this was a qualitative, investigative study. If compounds were not observed, increasing time and temperature to match the intended shelf life under normal storage conditions, e.g., 65 days at 50 °C simulating 12 months at 25 °C, may increase concentration into a detectable range more representative of the long-term simulated leachable study especially if the source of the compound is derived from the CCS.

**FIGURE 2:**
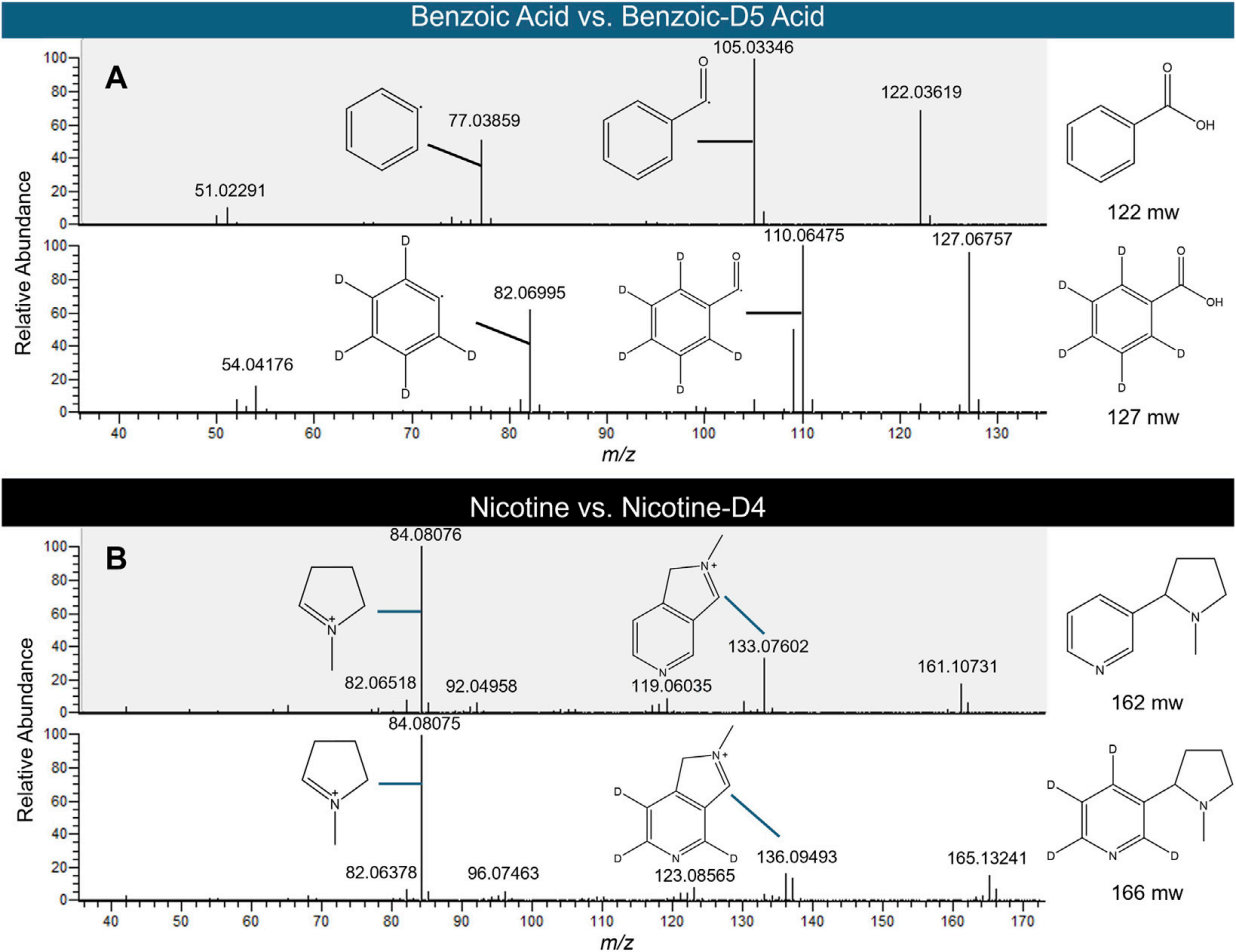
GC-MS spectra of benzoic acid and benzoic-D5 acid **(A)** and nicotine and nicotine-D4 **(B)** in the unlabeled and labelled e-liquid samples.

**FIGURE 3:**
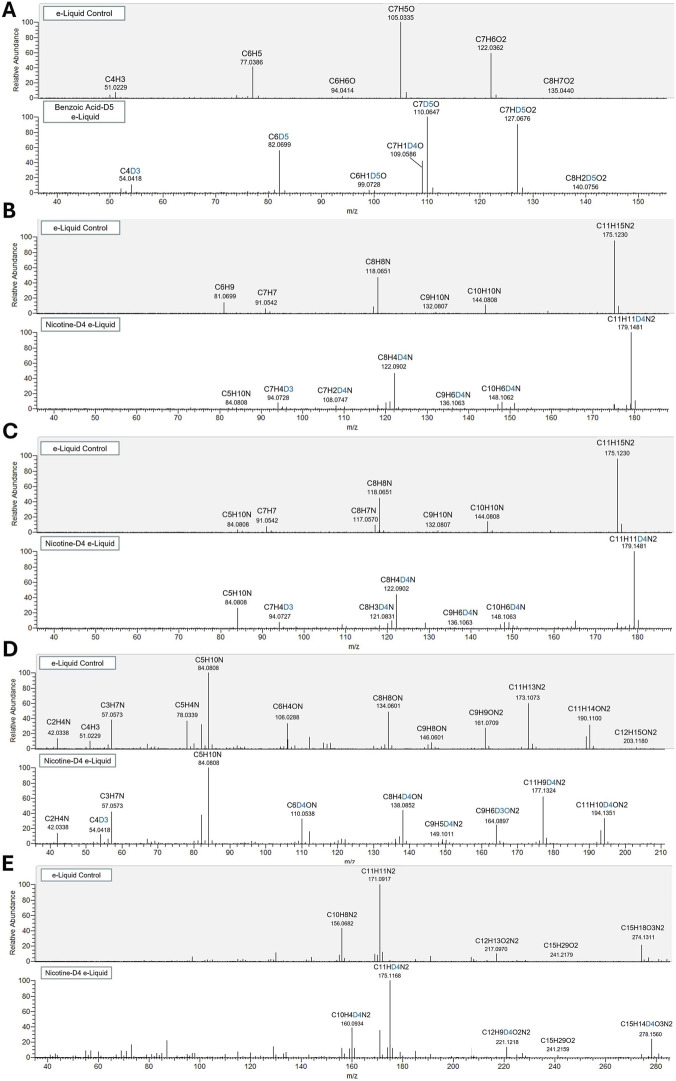
GC-MS spectra of Compounds **(A–E)** in e-liquid control (top) and isotopically labelled single ingredient e-liquid (bottom). Deuterium atoms are denoted in blue.

Since compounds A-E were generated outside the JUUL2 pod system, and therefore are not leachable compounds, isotopic labeling experiments were performed to determine the source. A total of four deuterated unflavored e-liquids were prepared, each containing an individual labelled primary ingredient (propylene glycol-D6, benzoic-D5 acid, nicotine-D4, and glycerin-D5) alongside the unlabeled e-liquid control, and were heated for 7 days at 50 °C. The tabulated results of the isotopic labeling experiment (Table 2) and mass spectra for compounds A-E (Figure 3) indicate the primary ingredient source for each reaction product. Spectral matches were confirmed based on high resolution measurements (<5 ppm mass accuracy), retention time, and fragmentation patterns. The results indicated that four of the five compounds were derived from nicotine, and one was derived from benzoic acid. Figure 2 shows the annotated mass spectra of benzoic acid compared to benzoic-D5 acid, and nicotine compared to nicotine-D4. In both cases, an expected mass shift of 5 Da and 4 Da for the signature fragments corresponded to the number of substituted deuterium atoms on the benzene and pyridine ring, respectively. It is worth noting for nicotine and nicotine-D4, the base peak observed at 84 *m/z* does not show a mass shift of 4 Da, as it is well known that this corresponds to the pyrrolidine ring that is unlabeled (Duffield A.M. et al., 1965). Additionally, the 136 *m/z* fragment from nicotine-D4 only contains three deuterium atoms, as it is a rearrangement involving the pyridine ring system (Duffield A.M. et al., 1965). Since both benzoic acid and nicotine contain labelled aromatic ring systems that remain intact upon electron impact fragmentation, mass shifts of 5 Da for compound A and 4 Da for compounds B-E fragments can be readily observed in the deuterium labelled individual e-liquid samples when compared to the unlabeled e-liquid control (Figure 3).

**TABLE 2:** Deuterated e-liquid tracing results for investigated leachable compounds by GC-HRMS.

Compound ID	Relative retention time (mins.)^1^	Updated compound information	Source of degradant	Observed deuterated m/z values
Compound A	0.77	Benzoic acid reaction product RRT 0.77	Benzoic acid	77 + D5, 105 + D5, 135 + D5
Compound B	1.43	Nicotine reaction product RRT 1.43	Nicotine	91 + D3, 118 + D4, 175 + D4
Compound C	1.44	Nicotine reaction product RRT 1.44	Nicotine	91 + D3, 118 + D4, 175 + D4
Compound D	1.10	Nicotine reaction product RRT 1.10	Nicotine	78 + D4, 84, 106 + D4, 134 + D4, 161 + D3, 173 + D4, 190 + D4
Compound E	1.46	Nicotine reaction product RRT 1.46	Nicotine	156 + D4, 171 + D4, 217 + D4, 274 + D4

1 Calculated based on the retention time of BHT.

**FIGURE 4:**
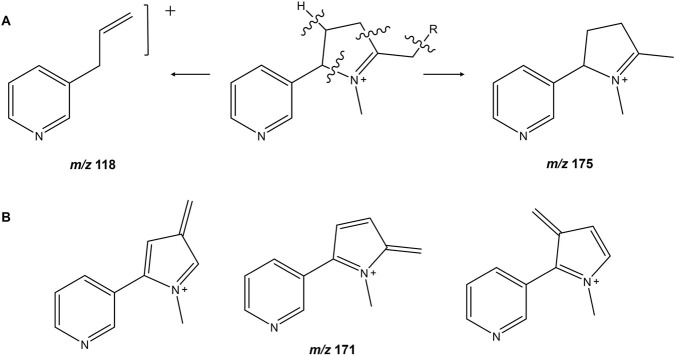
Predicted fragmentation scheme of Compounds B and C for ions 118 and 175 *m/z* **(A)**. Potential structures for fragment ion 171 *m/z* observed in Compound E **(B)**.

In Figure 3, compounds A and D mass spectra were straightforward to interpret, and confirm the source, given the significant overlap observed between the fragmentation patterns of primary ingredients benzoic acid (77, 105, and 122 *m/z*) and nicotine (78 and 84 *m/z*) and expected mass shifts. However, compounds B, C and E may not be as easily interpreted, and therefore, observed fragment peaks merit additional explanation. Compounds B and C are similar in retention time with nearly identical mass spectra, with two prominent fragments at 118 and 175 *m/z*, suggesting these two compounds are isomers. These fragments had been detected in a previous study while investigating nicotine and propylene glycol reaction products (Yan et al., 2021). Though the same molecular formula (C11H15N2) was predicted for 175 *m/z* for Compound B and C, the 118 *m/z* fragment composition (C8H8N) differed. Figure 4A shows the predicted fragmentation scheme for compounds B and C based on the mass spectra shown in Figure 3. The observation of 175 and 118 *m/z*, both fully labelled in the deuterated nicotine e-liquid, suggest the nicotine reaction products are substituted at the C(4) atom of the pyrrolidine ring. Based on the nitrogen rule, a compound with an even number of nitrogen atoms will have an even molecular ion (Gross J. H., 2017), however, the molecular ion was not observed for either compound B or C. This is common due to electron impact being a hard ionization technique, especially at a negative bias voltage of 70 eV, and the compounds were present in low abundance. Additional structural information could not be predicted, designated with “R” in Figure 4A. For compound E, 171 (C11H11N2) and 156 (C10H8N2) *m/z* both contained intact deuterium atoms located on the pyridine ring, and had similar predicted molecular formulae as the 175 *m/z* fragment in compounds B and C. Figure 4B shows proposed structures for the 171 *m/z* fragment, however, the exact location could not be predicted based on the number of possible structures plausible for the larger fragments associated with E, 217 and 274 *m/z*. Regardless of the substituent location on the pyrrolidine ring, the observed 156 *m/z* fragment was attributed to the loss of the CH3 group attached to the nitrogen in the pyrrolidine ring.

Although the structures of compounds A-E were not fully elucidated, their source was able to be attributed to primary e-liquid ingredients. The estimated concentrations of each of the four nicotine related reaction products shown in Table 1 were less than 5 mcg per pod. For a single unflavored JUUL2 pod utilized in this study, 5.0% nicotine is equivalent to approximately 65,000 mcg/pod, based on approximately 1.30 g e-liquid capacity. The estimated concentrations of compounds B-E corresponded to less than 0.05% of the total nicotine concentration used in the e-liquid formulation analyzed for the simulated leachable study. While there currently is no formal guidance for nicotine impurities in e-cigarettes, the USP monograph for pharmaceutical grade nicotine stipulates no more than 0.1% for unspecified nicotine impurities (USP, 2016). Reported, estimated concentrations of compounds B-E were 10 times lower than USP specifications for unspecified nicotine compounds. It is well understood that nicotine degrades over time primarily into known degradants such as β-nicotyrine, cotinine, myosmine and nicotine-N-oxide (Etter et al., 2013), and thus USP has included limits for these specific compounds in pharmaceutical grade nicotine. More importantly, Table 1 shows minimal differences between the estimated concentrations reported in the e-liquid and aerosol generated by the JUUL2 System using two puffing regimes. This confirms that the aerosolization process does not further increase the concentration of the observed e-liquid reaction products, which is significant because ENDS users are exposed to generated aerosol and not e-liquid. One of the five compounds, Compound E, was not observed in aerosol, indicating it may thermally degrade upon aerosolization or does not transfer to the aerosol; however, additional investigations would be needed to substantiate these hypotheses.

While the findings of this study offer valuable insights into the source of partially identified organic compounds in long-term leachable studies on ENDS, they should also be contextualized by a few limitations. Williams and Talbot (2019), Turner et al. (2024) and Pasiecznik et al. (2026) provide photographs of the diverse materials used in the construction of ENDS products over the past decade—starting with first generation cig-a-like products, to more modern disposable products. Because of the diverse materials used in the manufacturing of ENDS products, the JUUL2 pod utilized in this study is not representative of the entire commercial ENDS market. Another limitation was that the experiments were designed to provide a quick, i.e., minimal replicates, qualitative approach through accelerated aging of unflavored e-liquid to investigate observed compounds outside the JUUL2 pod. Compounds A-E formed after simulation of only 11 days of ambient storage in a vial which confirms that the CCS most likely is not required to promote the formation of the observed compounds in formulation contained within the CCS, however, additional studies would be needed to understand the CCS role on the concentration as this was not a quantitative study. The accelerated aging was performed by controlling two variables, time and temperature, while the headspace was uncontrolled, but a minimum of two times larger than the sample volume was maintained in the study (i.e., 1 mL of sample with at least 2 mL of headspace). Because four out of the five observed compounds were nicotine related, and nicotine is susceptible to hydrolytic and oxidative degradation (Bansal et al., 2018), the water and oxygen content in the headspace of the experiments may play a role in the formation of the observed compounds. Although the impact of headspace on reaction products observed in this study is a limitation, the reaction kinetics, formation and further structural elucidation of small nicotine and benzoic acid related organic compounds are out of the scope of extractable and leachable studies. It is critical to understand that NTA reporting, despite the use of controls and specific workflows to isolate CCS related compounds, can be subjective depending on the laboratory, method (including sample preparation, instrumental analysis, and data processing), and sample matrix expertise (Nason et al., 2025). This case study is an excellent example of the aforementioned sentiment: a third-party laboratory conducted a routine NTA leachable study using proprietary methods and data processing techniques on the unflavored e-liquid removed from the JUUL2 pod. The study e-liquid was compared to a control, which was the same unflavored e-liquid not stored in the JUUL2 pod. Per internal quality assurance and quality control measures, and no observation of the compounds in the blank, compounds A through E met criteria for reporting as a partially identified alkylbenzene or alkylbenzeneamine related compound and did not meet criteria to report as nicotine or benzoic acid related. This outcome was to be expected because third-party extractables and leachables testing laboratories are not specialized in e-liquid analysis, and investigation into the source of all organic compounds observed in a leachable study is typically out of scope in routine analysis. Lastly, one unflavored e-liquid pertinent to JUUL2 formulations was utilized for this study. Similar to the diversity of materials used in construction of ENDS products, primary ingredients used in commercial ENDS formulations are also diverse in the PG:VG ratio (Smith et al., 2024), amount of nicotine, and type of acid additive (Harvanko et al., 2020; Pappas et al., 2024), all of which affect the formation of ingredient-related reaction products. More specifically, almost no unflavored e-liquids are commercially available, and extrapolation of results is limited as it is well known that flavor ingredients can react in e-liquid prior to aerosolization (Erthyropel et al., 2019; Miller IV et al., 2025; Page et al., 2025) which may enhance or sequester the formation of primary ingredient related reaction products.

## 4 Conclusion

This work presents an analytical approach investigating the origin of potential organic leachable compounds reported in long-term simulated leachable studies for the JUUL2 pod, a closed-pod based ENDS product. In summary, the isotopic labelling experiments successfully traced the source of five partially identified organic compounds reported in leachable studies and confirmed their relation to primary ingredients instead of the JUUL2 pod. The need for additional resources regarding the reporting of E&L studies for ENDS manufacturers is critical, as leachables are one of three potential sources of genotoxic constituents per U.S. FDA memorandum (2024) applicable to PMTA reporting for marketing ENDS products in the United States. Due to the potential health risks associated with exposure to metals in aerosol, a tremendous focus of ENDS E&L research has been primarily centered on inorganic materials leaching from the heating element (Gong et al., 2023). However, organic compounds have the potential to leach from the CCS and other components (e.g., gaskets, seals, etc.) that are in contact with the e-liquid formulation for the duration of the shelf-life of ENDS products prior to use (Wei et al., 2020). The potential exposure and subsequent risk to organic leachables to ENDS users is the basis for the aforementioned U.S. FDA memorandum (2024). Therefore, the approach described herein is most applicable for ENDS manufacturers that currently have limited guidance and techniques for studying organic extractable and leachable compounds in ENDS products. Similar to E&L assessments for drug products and medical devices (Street et al., 2025), providing information regarding the origin of observed partially identified organic compounds in non-targeted screening E&L methods shown in this work allows toxicologists to appropriately categorize and contextualize the overall risk associated with the use of materials used in the construction of ENDS products to ensure a comprehensive safety assessment is conducted.

## Data Availability

The original contributions presented in the study are included in the article/supplementary material, further inquiries can be directed to the corresponding authors.
